# Complement C3 deposition restricts the proliferation of internalized *Staphylococcus aureus* by promoting autophagy

**DOI:** 10.3389/fcimb.2024.1400068

**Published:** 2024-09-06

**Authors:** Yining Deng, Yunke Zhang, Tong Wu, Kang Niu, Xiaoyu Jiao, Wenge Ma, Chen Peng, Wenxue Wu

**Affiliations:** ^1^ National Key Laboratory of Veterinary Public Health, Animal Disease Diagnostic Laboratory, College of Veterinary Medicine, China Agricultural University, Beijing, China; ^2^ College of Veterinary Medicine and Biomedical Sciences, Texas A&M University, College Station, TX, United States; ^3^ Institute of Laboratory Animal Sciences, Chinese Academy of Medical Sciences & Peking Union Medical College, Beijing, China; ^4^ Biotechnology Research Institute, Chinese Academy of Agricultural Sciences, Beijing, China

**Keywords:** complement C3, *Staphylococcus aureus*, autophagy, ATG16L1, intracellular proliferation

## Abstract

Complement C3 (C3) is usually deposited spontaneously on the surfaces of invading bacteria prior to internalization, but the impact of C3 coating on cellular responses is largely unknown. *Staphylococcus aureus* (*S. aureus*) is a facultative intracellular pathogen that subverts autophagy and replicates in both phagocytic and nonphagocytic cells. In the present study, we deposited C3 components on the surface of *S. aureus* by complement opsonization before cell infection and confirmed that C3-coatings remained on the surface of the bacteria after they have invaded the cells, suggesting *S. aureus* cannot escape or degrade C3 labeling. We found that the C3 deposition on *S. aureus* notably enhanced cellular autophagic responses, and distinguished these responses as xenophagy, in contrast to LC3-associated phagocytosis (LAP). Furthermore, this upregulation was due to the recruitment of and direct interaction with autophagy-related 16-like 1 (ATG16L1), thereby resulting in autophagy-dependent resistance to bacterial growth within cells. Interestingly, this autophagic effect occurred only after C3 activation by enzymatic cleavage because full-length C3 without cleavage of the complement cascade reaction, although capable of binding to ATG16L1, failed to promote autophagy. These findings demonstrate the biological function of intracellular C3 upon bacterial infection in enhancing autophagy against internalized *S. aureus*.

## 1 Introduction

The complement system is an indispensable component of the immune system. It contributes to the recognition and elimination of various types of pathogenic microorganisms, including bacteria (Joiner et al., 1984). Complement component 3 (C3) accounts for the vast majority of the complement proteins circulating in the blood, representing approximately 70–80% of all complement proteins (Ricklin et al., 2010). When exposed to invading bacteria, the complement system is activated, and C3 is hydrolyzed spontaneously, leading to subsequent deposition of C3 on bacterial surfaces (Pangburn and Muller-Eberhard, 1983). C3 deposition (which is enabled by thioester interactions) drives various biological processes, including activation of downstream effector proteins, formation of the membrane attack complex (MAC), activation of the inflammatory responses, and enhancement of bacterial phagocytosis (Ricklin et al., 2016).

*Staphylococcus aureus* is a Gram-positive bacterium that causes many diseases in humans and animals. This microorganism has garnered significant attention owing to the emergence and widespread dissemination of antibiotic resistance and the high mortality rate (10% to 30%) associated with the bloodstream infections it causes (Cosgrove et al., 2003). Traditionally, *S. aureus* is considered to be a facultative extracellular bacterium. Emerging evidence suggests that *S. aureus* can invade and survive within certain types of immune cells (e.g., neutrophils, macrophages), thereby allowing it to evade surveillance by the immune system to cause persistent infections (Kubica et al., 2008; Rigby and DeLeo, 2012). During bacterial invasion, most bacterial cells (including *S. aureus*) that enter host cells are deposited with activated C3 (Thomer et al., 2016). However, the role of C3 coating on bacterial surfaces, particularly how C3-coated bacteria interact with and modulate innate immune responses, remains elusive.

Autophagy is an evolutionarily conserved cellular process responsible for protein degradation and can be induced in cells by various stimuli, including pathogenic invasion [9]. Bacteria-stimulated autophagy is typically a host defense mechanism against an infection but can also support bacterial survival in certain circumstances (Zheng et al., 2022). When pathogens induce autophagy, nascent phagophores form within the cytoplasm, and gradually mature to form a closed and double membrane-surrounded vesicle named autophagosome (Randow and Youle, 2014). Subsequently, autophagosomes fuse with lysosomes to initiate protein degradation, leading to clearance of invading bacteria (Xie and Klionsky, 2007). Autophagy is induced when *S. aureus* activates pattern recognition receptors (PRRs) such as toll-like receptor 2 (TLR2) and nucleotide-binding oligomerization domain-containing protein 2 (NOD2), and is considered as a defense mechanism against bacterial infection (Askarian et al., 2018; Wang et al., 2021). Nevertheless, *S. aureus* evolved to develop mechanisms to evade from autophagy-mediated degradation by expressing bacterial proteins that also function as virulence factors (Cai et al., 2020). For example, α-toxin and iron-regulated surface determinant protein B (IsaB) secreted by *S. aureus* is able to prevent the maturation of autophagosomes and its fusion with lysosomes (Liu et al., 2015; Maurer et al., 2015). Without the active enzymes provided by lysosomes, the autophagosomes become perfect niches for *S. aureus* to survive and proliferate within cells (Weiss and Schaible, 2015). Autophagy-related 16-like 1 (ATG16L1) is characterized as a subunit of ATG12-ATG5/ATG16 complex which plays a crucial role in LC3 lipidation and autophagosome formation (Cadwell et al., 2008). Studies showed that ATG16L1 interacts with C3 positive serum (C3^+ive^) and drives autophagic restriction of *Listeria* replication (Sorbara et al., 2018). As C3 is found to be deposited on the surface of *S. aureus* prior to cellular invasion, whether C3 participates in autophagic activation is a fascinating question. We hypothesized that the C3 coating functions as an “intracellular chaperone” to promote autophagic flux upon *S. aureus* infection.

In this study, we showed that *S. aureus* with C3 deposition (C3^+ive^-*S. aureus*) could invade and activate autophagy in both phagocytes (e.g., THP-1 cells and RAW264.7 cells) and non-phagocytes (e.g., A549 cells and MDBK cells) of different species. The deposited C3 directly interacted with ATG16L1 and promoted autophagy. This process necessitated C3 opsonization, resulting in an ATG16L1-dependent restriction of *S. aureus* proliferation. Taken together, our results demonstrated that the C3 coatings contributed to enhanced autophagy induced by bacterial invasion through recruiting and binding to ATG16L1, which protected host cells from bacterial proliferation.

## 2 Materials and methods

### 2.1 Cell culture

A549 (American Type Culture Collection Manassas, VA, USA, catalog number: ATCC CRM-CCL-185), RAW264.7 (ATCC TIB-71), MDBK (ATCC CCL-22), and 293T (ATCC CRL-3216) cells were maintained in Dulbecco’s modified Eagle’s Medium (DMEM) supplemented with sodium pyruvate, L-glutamine, 10% heat-inactivated fetal bovine serum (FBS) and 1% penicillin–streptomycin solution in a humidified incubator with 5% CO_2_. THP-1 cells (ATCC TIB-202) were grown in RPMI 1640 medium containing L-glutamine, sodium pyruvate, 1% HEPES, supplemented with non-essential amino acids, and incubated as described above.

### 2.2 Complement opsonization

Luria–Bertani broth was used to cultivate *S. aureus* (ATCC 29213) at 37°C overnight with agitation (200x rpm). They were used in the mid–late log phase at an optical density (OD) calculated at a wavelength of 600 nm of 1.0. Bacteria (500 μL) were washed 3 times with 1x PBS. Next, 20% C5-depleted (C3^+ive^) or C3-depleted (C3^-ive^) human serum (Complement Technology) was added to bacteria, the mixture was incubated at 37°C for 30 min for complement opsonization, every 10 min, vortexed the mixture for 30s to mix bacteria and serum thoroughly. The bacteria were rinsed three times with 1x PBS after opsonization to eliminate any unbound complement components. They were then resuspended in serum-free DMEM to be used for infection.

### 2.3 Bacterial quantification and infection

For constructing infection models and autophagy detection, the input multiplicity of infection (MOI) for A549 cells was 20 or 100, depending on the test. The input MOI for THP-1, MDBK, and RAW264.7 cells was 50. In intracellular-replication experiments, A549 and RAW264.7 were infected at a MOI of 20. After inoculum had been added to cells in serum-free DMEM, plates were spun at 500 × *g* in a centrifuge (Allegro; Beckman Coulter, Fullerton, CA, USA) for 5 min at room temperature. Cells with bacteria were incubated at 37°C for 1 h and then washed thrice with PBS. DMEM supplemented with 10% FBS and gentamycin (100 μg/mL) was added to inhibit the survival of extracellular bacteria.

In intracellular-replication experiments, before being lysed in 0.1% Triton X-100, infected cells were rinsed thrice with PBS. After being serially diluted, the lysates were applied to LB agar plates. The latter were inverted overnight in an incubator at 37°C, and bacterial colonies were counted and recorded.

### 2.4 Cell viability and cell counting kit-8 assay

Experimental procedures were carried out according to the instructions provided on the CCK-8 kit (Beyotime Institute of Biotechnology). Cells were seeded at 2 × 10^3^ cells/well into 96-well plates and incubated for 24 h following infection with *S. aureus* opsonized with C5-depleted serum (C3^+ive^), C3-depleted serum (C3^-ive^), human normal IgG, or PBS. At different time points (0, 2, 4, 6, and 8 h), CCK-8 reagent (10 μL) was added to the complete medium (100 μL) for further incubation. Subsequently, the OD_450_ was measured by a plate reader (Multiskan FC; Thermo Scientific). Cell viability was converted from OD_450_ using the formula [(As − Ab)/(Ac − Ab)] × 100%, where AS is the absorbance of experimental wells, Ac is the absorbance of control wells, and Ab is the absorbance of blank wells.

### 2.5 Western blot

Prior to protein extraction, cells were rinsed with ice-cold PBS. The total protein was extracted with a cell lysis buffer for western blotting and immunoprecipitation supplemented with phenylmethylsulphonyl fluoride (1 mM; Beyotime Institute of Biotechnology). Bicinchoninic acid assay (BCA) (Thermo Scientific) was used to quantify the protein content and standardize protein loading across samples according to the manufacturer’s directions. Following dissolving lysates in 6× protein-loading buffer (TransGen Biotech) and heating at 100°C, proteins were resolved on 10%–18% polyacrylamide gels (PAG) and transferred to polyvinylidene difluoride (PVDF) membranes. The latter were blocked in blocking buffer, which is 5% non-fat milk in Tris-buffered saline (TBS) with 0.1% Tween 20 (TBST) for 1 h at room temperature and incubated with primary antibodies at 4˚C. Goat anti-C3 (Complement Technology, catalog #: A213), rabbit anti-ATG16L1 (Cell Signaling Technology, catalog #: 8089), rabbit anti-LC3A/B (Millipore Sigma, catalog #: SAB5700812-100UL), and rabbit anti- p62/SQSTM (Millipore Sigma, catalog #: P0067-200UL) were used at 1:1000 dilution in blocking buffer. Mouse anti-myc (Abcam, catalog #: ab289980), mouse anti-glyceraldehyde 3-phosphate dehydrogenase, horseradish peroxidase (HRP) conjugated (GADPH-HRP; Beyotime Institute of Biotechnology, catalog #: AF2823-50μl), and mouse anti-β-actin, HRP conjugated (Beyotime Institute of Biotechnology, catalog #: AF2815-50μl) were used at 1:2000 dilution. On the next day, PVDF membranes were washed thrice with TBST and incubated with horseradish peroxidase-conjugated secondary antibodies (Beyotime Institute of Biotechnology) diluted at 1:10000 in blocking buffer for 1 h at room temperature and detected with ECL Prime (Thermo Scientific). Gray intensity of bands was analyzed by densitometric of Image-J.

### 2.6 Immunofluorescent microscopy

Cells were seeded on coverslips (Solarbio Life Science) at desired density. After treatment, they were fixed with 4% paraformaldehyde (PFA) for 15 min, and permeabilized with 0.1% triton X-100 for 20 min at room temperature after gently washed with iced-old PBS for 3 times. Then, 2% bovine serum albumin (BSA) in 1 × PBS was used to block the cells for 1 h at room temperature. After 3-time washes by PBS, the cells were incubated by primary antibody (1:100 dilution in 2% BSA) at 4°C overnight or at room temperature for 1 h. After 3-time washes, cells were incubated with secondary antibody conjugated with fluorophore (1:500 dilution in 2% BSA) for 1 h at room temperature. Use 1 × PBS containing 0.1% triton X-100 (PBST) to wash cells for 2 times, then add Hoechst 33342 stain (Thermo Scientific) for 5 min at room temperature. Again, use PBST to remove the unbound stains for 3 times. Cells were observed and acquired by confocal microscope Nikon AX/AX R with the microscope objectives of 10x/0.45 NA, 40x/0.95 NA, 60x/1.40 NA Oil, and 100x/1.45 NA Oil were used in this manuscript. Images were processed and analyzed by NIS-Elements Viewer software and Image-J.

### 2.7 shRNA knockdown

Lentivirus expression vector stocks were generated by transduction, using plasmids pMDG gag-pol, pRSV–Rev, pVSV–G Env, and the pLKO.1 *ATG16L1* shRNA (laboratory-retained, used as mentioned (Zhu et al., 2023)) to co-transfect 293T cells. The ATG16L1 gene was knocked down by the complementary sequence: 5’- CCGGGTCATCGACCTCCGGACAAATCTCGAGATTTGTCCGGAGGTCGATGACTTTTTG-3’ in the pLKO.1 *ATG16L1* shRNA. Non-targeting control was obtained from SIGMA MISSION^®^ TRC-Hs 1.0 (Human). After 48 h, supernatants were collected, centrifuged, and filtered to remove cell debris. A549 cells or RAW264.7 cells were transduced with the respective lentiviruses or empty vector control lentivirus for 72 h as described previously (Xu et al., 2019). This action was followed by selection with puromycin (2 μg/mL), which was determined to kill about 95%–99% of untransduced cells. Surviving cells were amplified, and knockdown was confirmed by western blotting.

### 2.8 Transfection and plasmids

A549 cells and 293T cells were cultured to ~60% confluency and transfected with the pCMV-myc plasmid containing full-length human C3 cDNA (National Center for Biotechnology Information (NCBI) reference sequence: NM_000064.4) or human ATG16L1 cDNA (NCBI reference sequence: NM_198890.3) or empty vector using jetPRIME™ (Polyplus Transfection, Illkirch-Graffenstaden, France) according to manufacturer’s instructions. Transfection efficiency was between 70% and 80% as determined with a ZsGreen-producing or mCherry-producing control vector.

### 2.9 Immunoprecipitation assay

The 293T cells in 10-cm dishes were grown to 60% confluency and transfected with pCMV-myc-C3-ZsGreens and/or pCMV-myc-ATG16L1-mCherry by jetPRIME™ (Polyplus Transfection). After 24 hours, cells were washed with ice-cold PBS and lysed with cell lysis buffer for Western blotting and immunoprecipitation (BIB) analysis. Cell lysates were centrifuged at 14,000 × *g* for 5 min at 4˚C and precleared with normal rabbit IgG and protein A+G agarose (BIB) at 4˚C for 1 h with rotation to remove nonspecific binding. Concerning the mixture of cell lysates and C3^+ive^-*S. aureus*, the collected supernatants after lysis were incubated with C3^+ive^-*S. aureus* directly for 2 h to remove nonspecific binding. Protein samples were centrifuged at 2,500 rpm for 5 min at room temperature. Then, the supernatant was collected and incubated with rabbit anti-C3 primary antibody or rabbit anti-ATG16L1 primary antibody and protein A+G agarose (BIB) at 4˚C overnight with rotation. Then, the A+G agarose was washed six times with lysis buffer. Bound proteins were eluted by boiling with 6× protein-loading buffer (TransGen Biotech) for 10 min. Proteins were resolved on 8%–15% polyacrylamide gels (BIB) and analyzed by Western blotting analysis with antibodies for C3 and ATG16L1.

### 2.10 Transmission electron microscopy

A549 cells were infected with *S. aureus* or C3^+ive^ *S. aureus* at 20 CFU/cell. After 4 h, cells were washed with PBS, fixed with 2.5% glutaraldehyde, dehydrated, and embedded in plastic resin for viewing with a transmission electron microscope (HT7800; Hitachi, Tokyo, Japan).

### 2.11 Statistical analyses

Statistical analyses were carried out using GraphPad Prism 9 software. The number of biological replicates used herein was estimated based on a power analysis with the following assumptions: the standard deviation would be ~20% of the mean; *P* < 0.05 when the null hypothesis was false; the effect size (Cohen’s d) was between 1.0 and 2.0. Percent LC3-positive phagocytes or CFU counts were determined for significance with the unpaired parametric *t*-test for two groups, with ANOVA for multiple groups, and corrected for Student’s *t* test and *post hoc* Tukey’s multiple comparisons. Randomization or blinding was not undertaken in this study.

## 3 Results

### 3.1 Internalized C3^+ive^-*S. aureus* colocalized with LC3 in cells of different species

**Figure 1:**
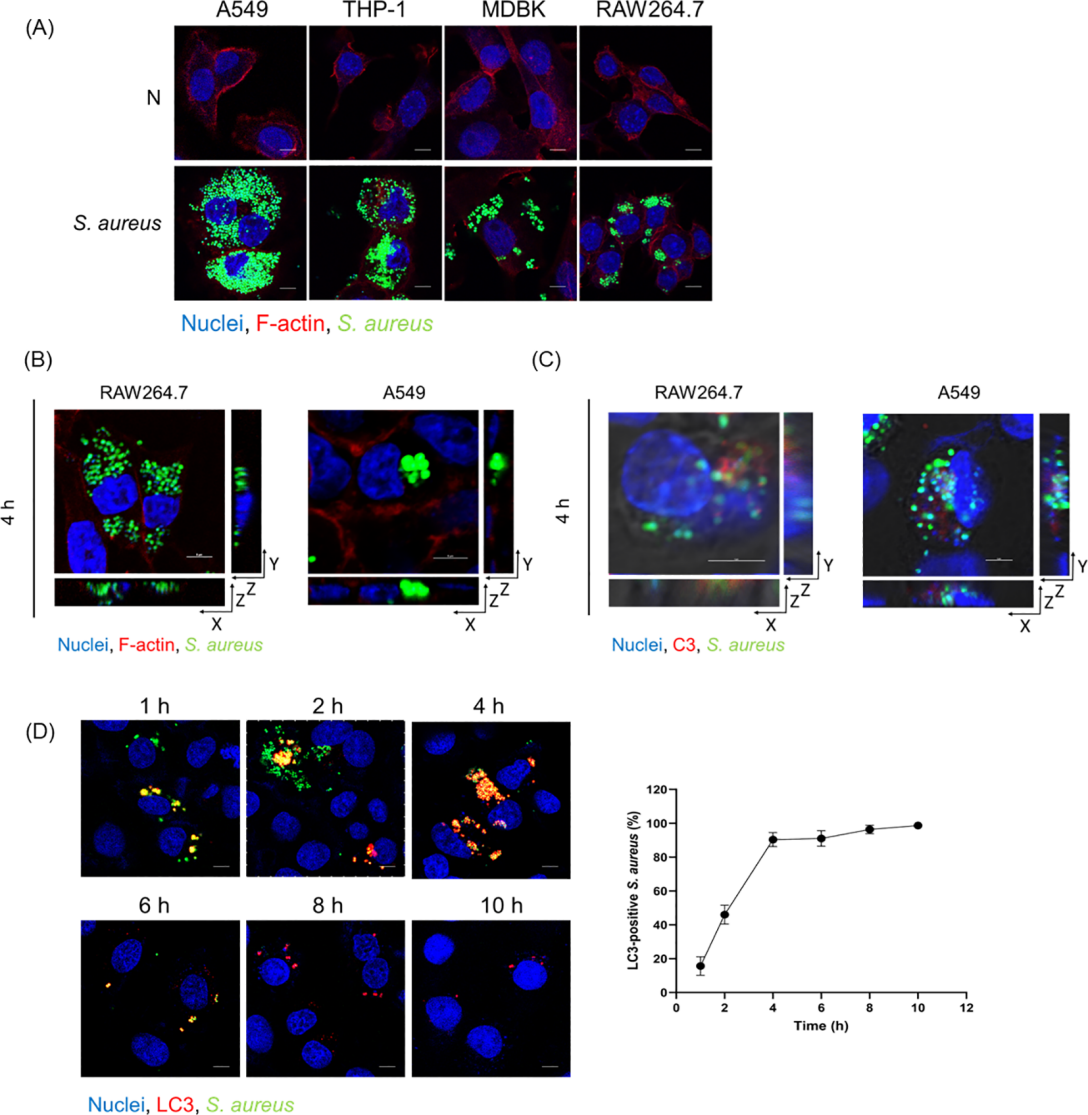
Internalized C3^+ive^-*S. aureus* induced autophagy. **(A)** Fluorescence images of internalized C3^+ive^-*S. aureus* in cells (A549, THP-1, MDBK, RAW264.7). Cells were infected with *S. aureus* at 100 CFU/cell prior to being fixed, permeabilized, blocked, and stained with phalloidin-rhodamine (excitation = 552 nm, emission = 565 nm) and Hoechst 33258 (excitation = 405 nm, emission = 454 nm), respectively. The scale bar was 5 µm. **(B, C)** A549 cells and RAW264.7 cells were infected with C3^+ive^-*S. aureus* at 100 CFU/cell. 3D images were taken in X-, Y-, and Z-axis sections, Z-axis section was cut every 1 µm or 2 µm. **(B)** F-actin were stained with phalloidin-rhodamine. **(C)** C3 deposition were stained with antibodies to C3 (excitation = 552 nm, emission = 565 nm). **(D)** A549 cells were infected with C3^+ive^-*S. aureus* at 20 CFU/cell. At 1, 2, 4, 6, 8. 10 hpi, cells were stained with antibodies to LC3 (excitation = 552 nm, emission = 565 nm). The scale bar was 5 µm. The proportion of *S. aureus* colocalized with LC3 was counted and analyzed at the corresponding time points from n = 3 independent experiments.

To test the function of C3 in autophagy regulation, we used human C5-depleted (C3^+ive^) serum, which allowed C3 deposition but avoided MAC formation, to incubate with *S. aureus*. The *S. aureus* strain we used was modified to stably express green fluorescent protein (GFP), and the level of GFP was used as a marker for *S. aureus* infection (Li et al., 2021). To ascertain if *S. aureus* with C3 deposition (C3^+ive^-*S. aureus*) could be internalized in cells, A549, THP-1, MDBK, and RAW264.7 cells were infected at 100 CFU/cell. GFP and F-actin (stained by phalloidin-rhodamine) were observed within the cells at 4 hours post-infection (hpi), indicating C3^+ive^ -*S. aureus* was internalized regardless of the origin of species of the cells used, or whether the cells were of myeloid lineage (**Figure 1A**). Next, we chose a non-phagocytic cell type (A549) and a phagocytic cell type (RAW264.7) to examine if C3 remained attached to the surface of *S. aureus* within host cells. A549 and RAW264.7 cells were infected with C3^+ive^ -*S. aureus* at 100 CFU/cell, and three-dimensional (3D) images were taken by a confocal microscope. The results showed C3 colocalized with *S. aureus* within the cells, suggesting C3 was still bound with internalized *S. aureus* (**Figures 1B, C**). Internalized C3^+ive^-*S. aureus* was hypothesized to induce autophagy as the microtubule-associated protein light chain 3 (LC3), a marker of autophagosomes, was found colocalizing with C3^+ive^-*S. aureus* (**Figure 1D**). LC3 signals increased in a time-dependent manner, and nearly all bacterial particles were surrounded by autophagosomes at 4 hpi. These results confirmed that C3 deposition was internalized with bacteria and colocalized with autophagic machinery.

### 3.2 *S. aureus* and C3^+ive^ *-S. aureus* induced autophagy in A549 cells

**Figure 2:**
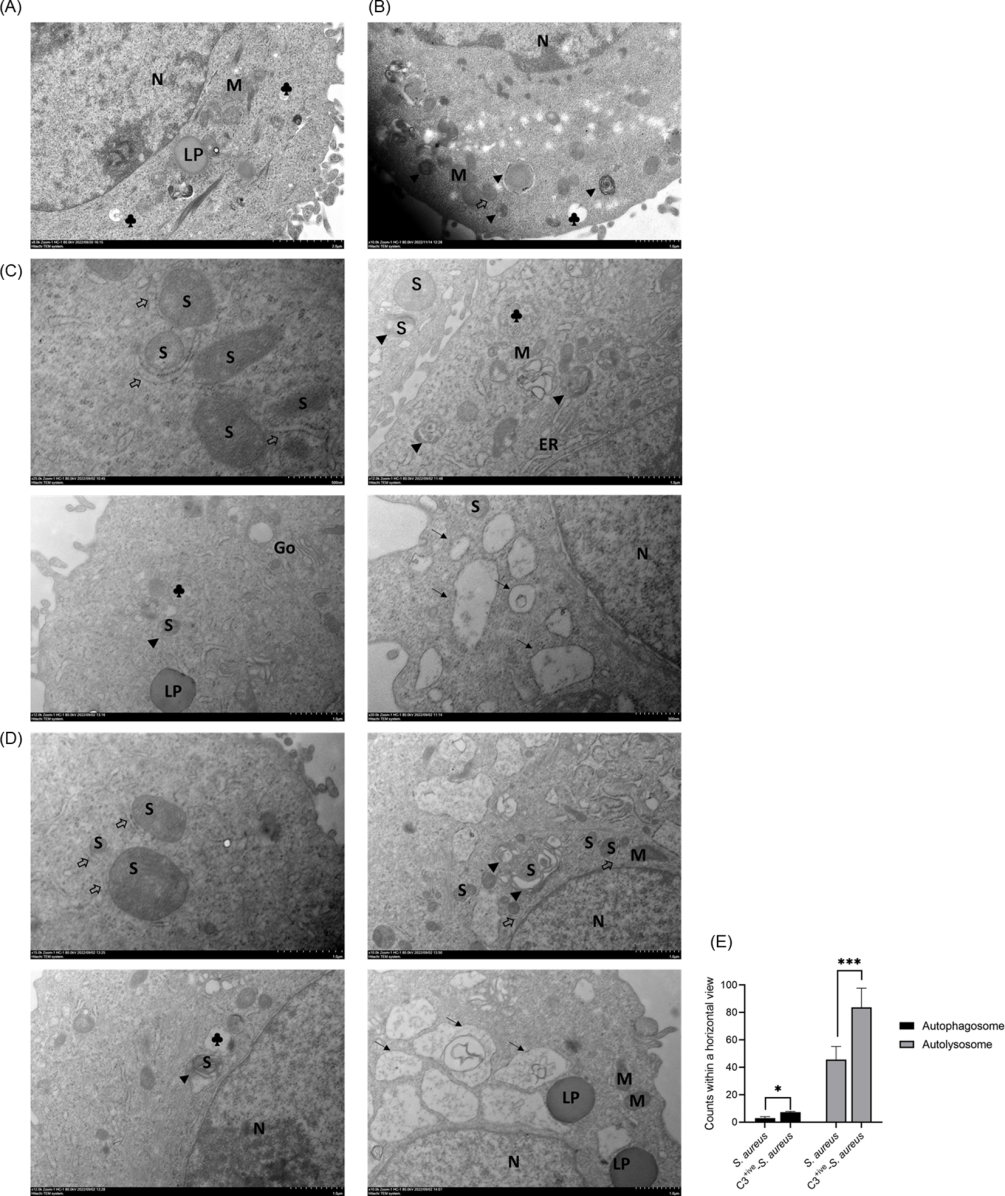
TEM of A549 cells with internalized *S. aureus* or C3^+ive^ *-S. aureus*. A549 cells were infected with 20 CFU/cell of *S. aureus* **(C)** or C3^+ive^ *-S. aureus* **(D)**. Cells treated **(B)** or not treated **(A)** with rapamycin (50 nM) were set as positive and negative controls, respectively. After 4 h, cells were fixed, sectioned, and analyzed by transmission electron microscopy. N, nuclei; M, mitochondria; S, *S. aureus*; ⇒, double-membrane; ▴, autophagosome; →, autolysosome; ♣, lysosome; Go, Golgi apparatus; ER, endoplasmic reticulum; LP: lipid droplet. Magnification factors are shown below each panel. **(E)** The number of bacteria in autolysosomes and autophagosomes within a horizontal view. Representative of n = 3 independent experiments. * and ***: *p≤0.5, ***p≤0.001.

Various autophagic components or processes, such as LC3-associated phagocytosis (LAP), contributes to defense against bacterial infection (Munoz-Sanchez et al., 2020). Studies have identified that phagocytosed *S. aureus* in neutrophils triggers LAP, and activates the formation of LC3-associated phagosomes (“LAPosomes”) with a single-membrane structure (Prajsnar et al., 2021). To gain a deeper understanding of the role of C3 on autophagy, transmission electron microscopy (TEM) was carried out to visualize the formation of autophagosomes in A549 cells with internalized *S. aureus* and C3^+ive^-*S. aureus* at 20 CFU/cell. If internalized bacteria mainly induced LAP, then LAPosomes with a single membrane would be formed in the cytoplasm, but autophagosomes with double a membrane were observed which confirmed that both *S. aureus* and C3^+ive^-*S. aureus* stimulated xenophagy. The typical submicroscopic structure of A549 cells was shown in **Figure 2A**. Cells treated with rapamycin induced autophagy and formed autophagosomes (marked with ▴) (**Figure 2B**). Consistent with the autophagosomes stimulated by rapamycin, *S. aureus* and C3^+ive^-*S. aureus* infection also drove the formation of autophagosomes in A549 cells. When internalized in the cytoplasm, *S. aureus* (**Figure 2C**) and C3^+ive^-*S. aureus* (**Figure 2D**) were positioned with double membranes (marked with ⇒), and were engulfed as the membrane elongated to form autophagosomes. The bacterial cells in autophagosomes were later degraded as the fusion of autophagosome and lysosome began to occur. These results suggest that *S. aureus* and C3^+ive^ *-S. aureus* induced autophagy as an antimicrobial strategy within A549 cells, whereas C3^+ive^ *-S. aureus* resulted in more autophagic vesicles (**Figure 2E**).

### 3.3 C3 deposition of *S. aureus* promoted autophagy

**Figure 3:**
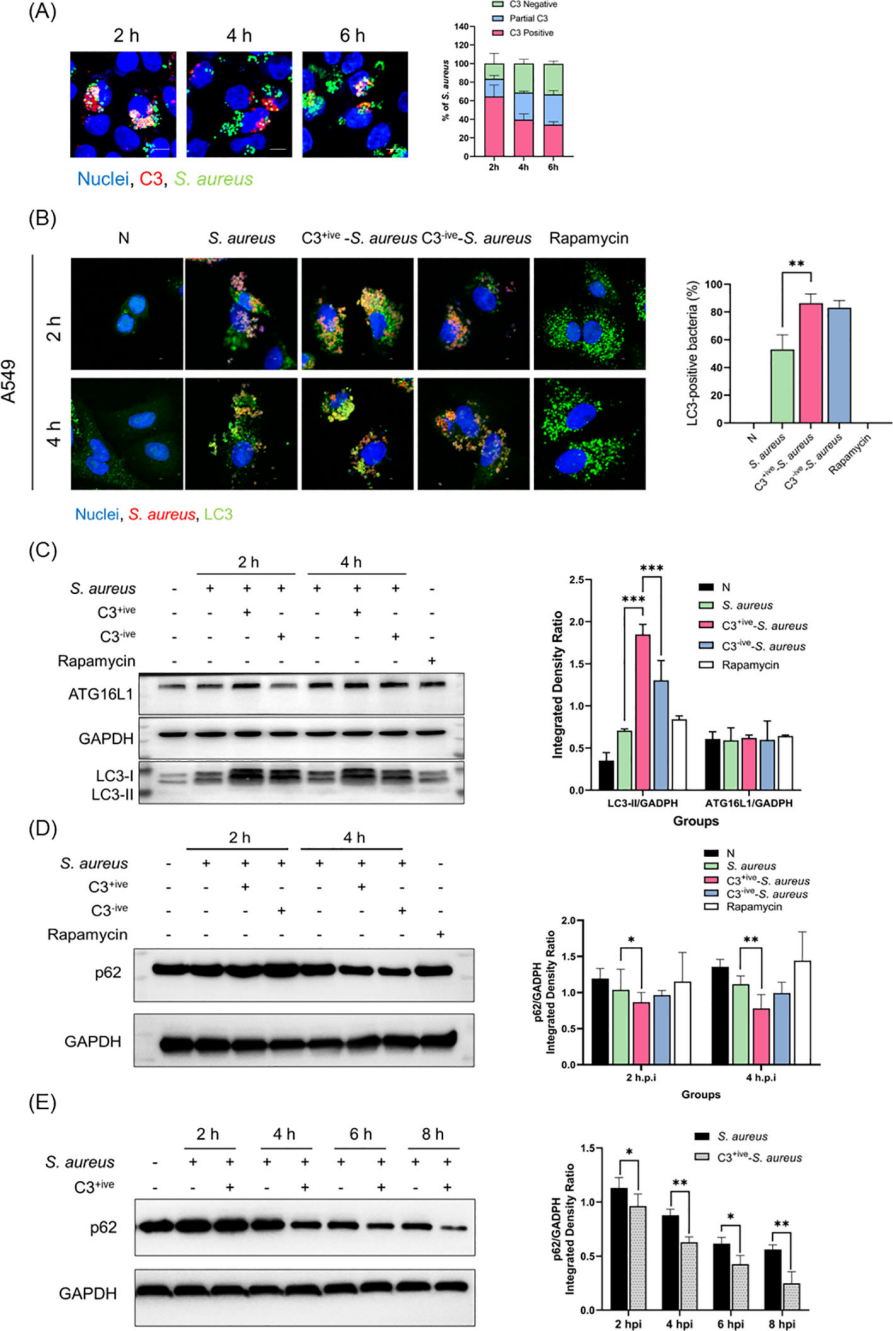
C3 deposition enhanced autophagic response. **(A)** A549 cells internalized with C3-opsonized *S. aureus* at 2, 4, and 6 hpi. The staining of C3 and nuclei is shown with antibodies and Hoechst 33258, respectively. The scale bar was 5 µm. Percentage of intracellular *S. aureus* that are positive for C3 at 2, 4, and 6 hpi. Representative of n = 2 independent experiments. **(B)** LC3 targeting in A549 cells at 2 and 4 hpi with *S. aureus*, C3^+ive^-*S. aureus* or C3^-ive^-*S. aureus*. Cells were stably expressed LC3-GFP (excitation = 488 nm, emission = 509 nm), and stained with antibodies to *S. aureus* (excitation = 552 nm, emission = 565 nm) after infection. The scale bar was 2 µm. Proportion of LC3 recruited by *S. aureus* was quantified from n = 3 independent experiments. **(C)** Western blotting showing expression of ATG16L1 and LC3 in A549 cells infected with *S. aureus*, C3^+ive^-*S. aureus* or C3^-ive^-*S. aureus*. Lysates from cells at 2 and 4 hpi were harvested and analyzed with antibodies to ATG16L1, LC3 and GADPH (loading control). Lines on both sides represent the electrophoretic positions and masses in kDa of marker proteins. All proteins were normalized to GADPH expression from n = 3 independent experiments. **(D)** Western blotting showing expression of p62 in A549 cells infected with *S. aureus*, C3^+ive^-*S. aureus* or C3^-ive^-*S. aureus* following by 0.5mg/mL Leupeptin treatment. P62 protein normalized to the expression level of GADPH, representative of n = 3 independent experiments. **(E)** Western blotting showing p62 expression in A549 cells infected with *S. aureus* opsonized with *S. aureus* or C3^+ive^-*S. aureus* at 2, 4, 6 and 8 hpi. P62 protein normalized to the expression level of GADPH, representative of n = 3 independent experiments. MOI = 100. *, ** and ***: *p≤0.5, **p≤0.01, ***p≤0.001.

The composition of serum is very complicated. To exclude the effect observed was caused by other non-C3 serum components, we used a C3-depleted serum (C3-negative, C3^-ive^) to incubate *S. aureus* (C3^-ive^-*S. aureus*) as a parallel control. *S. aureus* was grown to the exponential phase and re-suspended in phosphate-buffered saline (PBS) with 20% C5-depleted (C3^+ive^) or C3-depleted (C3^-ive^) serum. Incubated *S. aureus* was then used to infect A549 cells at 100 CFU/cell. Intracellular C3 was detected by immunofluorescence assay (IFA). We observed that C3 was localized to *S. aureus* following bacterial infection, most *S. aureus* were C3 positive or with partial C3 deposition at 2, 4, and 6 hpi (**Figure 3A**).

Endogenous LC3 was monitored in A549 cells by IFA at 2 and 4 hpi. There was a significant increase in the colocalization of C3^+ive^-*S. aureus* with LC3 compared to *S. aureus*, indicating that C3 contributed to LC3 targeting. Meanwhile, the C3^-ive^-*S. aureus* also caused more LC3 targeting than *S. aureus* alone, suggesting other serum ingredients may contribute to the process, although playing a less significant role than C3 (**Figure 3B**).

LC3-I to LC3-II conversion is a well-accepted marker for autophagic activation (Tanida et al., 2008). We monitored the levels of LC3-I and LC3-II and observed a greater conversion of LC3-I to LC3-II in C3^+ive^-*S. aureus*-infected cells than C3^-ive^-*S. aureus*-infected ones, suggesting the involvement of C3 in autophagic activation (**Figure 3C**). Also, compared with incubation with PBS, *S. aureus* incubated with 20% C3-depleted (C3^-ive^) serum facilitated LC3-I to LC3-II conversion, which was in accordance with our previous results.

SQSTM1/p62 protein (p62) is a cellular protein selectively wrapped into the autophagosomes and subsequently degraded by proteases in the autolysosomes (Yoshii and Mizushima, 2017). The expression of p62 is negatively correlated with autophagic flux and was also used as an autophagic marker (Liu et al., 2014). To further demonstrate the effect of C3 on autophagic flux, p62 was examined by Western blotting analysis. The level of p62 protein expression decreased upon *S. aureus* invasion, and C3 deposition exacerbated this decrease, indicating C3 upregulated autophagic flux (**Figure 3D**). To further confirm the role of C3 deposition on autophagic flux, p62 was measured in A549 cells after *S. aureus* and C3^+ive^-*S. aureus* infection at different time points after infection (2, 4, 6, 8 hpi) without Leupeptin inhibition. As shown in **Figure 3E**, p62 gradually reduced with the duration of infection, and C3^+ive^-*S. aureus* caused a more rapid reduction than *S. aureus*. Overall, these data demonstrated that C3 deposition of *S. aureus* promoted autophagic activation and accelerated the autophagic flux.

### 3.4 Bacteria-associated C3 recruited and interacted with ATG16L1

**Figure 4:**
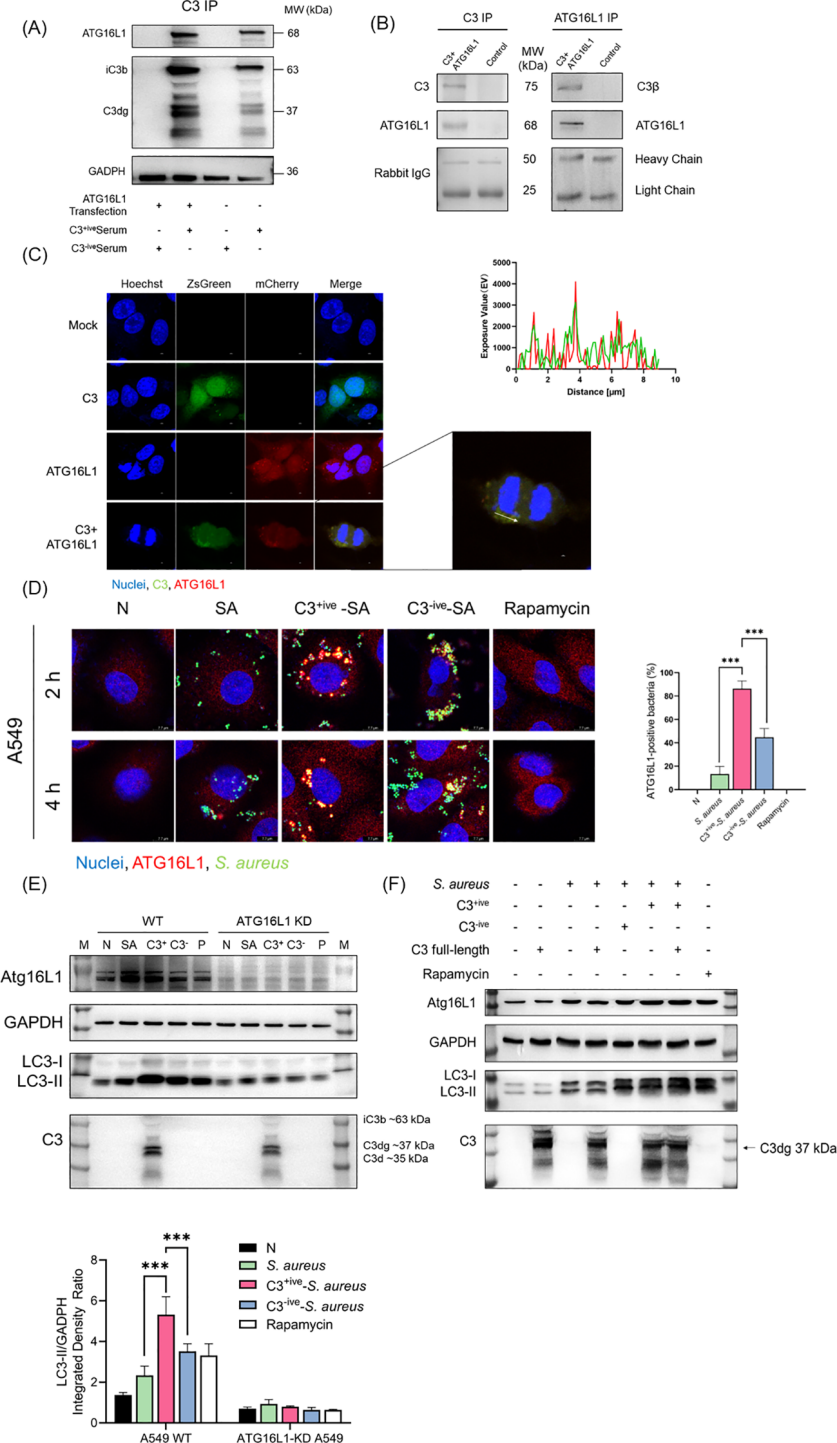
C3 recruited and interacted with ATG16L1. **(A)** C3 and ATG16L1 blots of immunoprecipitated proteins following C3 immunoprecipitation from lysates of A549 cells transfected with or without ATG16L1 in the presence or absence of C3^+ive^-*S. aureus* infection. Representative of n = 2 independent experiments. **(B)** C3, ATG16L1, and rabbit IgG blots of input and immunoprecipitated proteins following C3 immunoprecipitation or ATG16L1 immunoprecipitation from lysates of 293T cells transfected with ATG16L1 and C3 overexpressed vectors. Representative of n = 3 independent experiments. **(C)** Colocalization of C3 and ATG16L1 in A549 cells. C3 and ATG16L1 overexpression vectors were transfected into A549 cells. After 24 h, cells were fixed, permeabilized, blocked, and stained with primary antibodies to C3 and ATG16L1 followed by fluorescent-conjugated secondary antibodies. The scale bar was 2 µm. A similar exposure value of C3 and Atg16L1 was observed at the white arrow. **(D)** ATG16L1 targeting in A549 cells at 2 and 4 hpi with *S. aureus*, C3^+ive^-*S. aureus*, or C3^-ive^-*S. aureus*. Cells were stained with antibodies to ATG16L1. The scale bar was 7.7 µm. Proportion of ATG16L1 recruited by *S. aureus* was quantified from n = 3 independent experiments. **(E)** Expression of ATG16L1 and conversion of LC3-I to LC3-II in wild-type and ATG16L1-knockdown A549 cells was determined by western blotting. Wild-type and ATG16L1-knockdown A549 cells were infected with *S. aureus*, C3^+ive^-*S. aureus* or C3^-ive^-*S. aureus*, and harvested at 4 hpi. **(F)** Expression of ATG16L1 and conversion of LC3-I to LC3-II in A549 cells was determined by western blotting. A549 cells were transfected with or without C3 full-length overexpression vector and then infected with *S. aureus*, C3^+ive^-*S. aureus*, or C3^-ive^-*S. aureus*. After 4 h, cells were lysed and proteins analyzed by western blotting with antibodies to ATG16L1, LC3, C3, and GAPDH. MOI = 20. *** p ≤ 0.001.

Studies have shown that the autophagy-related protein, ATG16L1, interacted with the complement C3d fragment (Sorbara et al., 2018). As C3 deposition on the bacterial surface continues to undergo hydrolysis by Factor H and produce multiple fragments (C3b, iC3b, C3dg, C3d), we chose to further confirm whether C3 fragments could interact with ATG16L1 by immunoprecipitation (Gordon et al., 1988; Zarantonello et al., 2023). Total lysates of 293T cells overexpressing ATG16L1 were mixed with C3^+ive^-*S. aureus*. Following the immunoprecipitation of C3, ATG16L1 was found co-precipitated with C3 fragments (**Figure 4A**). To rule out artificial effect caused by protein overexpression, we examined the interaction of endogenous ATG16L1 with the overexpressed full-length C3 within cells by co-transfecting 293T cells and A549 cells with ATG16L1 and C3 overexpressed vectors. In 293T cells, C3β was found to pulldown ATG16L1 and the reciprocal IP showed the same results (**Figure 4B**). In addition, the full-length C3 colocalized with ATG16L1 according to confocal microscopy (**Figure 4C**). Taken together, these data demonstrated that ATG16L1 interacted and colocalized with full-length C3 and its cleaved products.

The higher level of autophagy induced by C3^+ive^-*S. aureus* was not accompanied by a change in ATG16L1 expression (**Figure 3C**). We hypothesized that ATG16L1 was recruited from dispersion by C3, which then controlled the location of the autophagosome and drove the conversion of LC3-I to LC3-II. A549 cells were infected with C3^+ive^-*S. aureus* and ATG16L1 were traced by immunofluorescence staining. Compared with *S. aureus* and C3^-ive^-*S. aureus*, C3^+ive^-*S. aureus* caused more aggregation of ATG16L1 (**Figure 4D**). To further confirm the regulatory role of ATG16L1 and C3 in autophagy, ATG16L1-knockdown cell lines were constructed using lentivirus-mediated short hairpin RNA (shRNA). C3 increased autophagy in wild-type A549 cells, but this increase disappeared in ATG16L1-knockdown A549 cells (**Figure 4E**). Correspondingly, compared with wild-type cells, LC3 expression was reduced in ATG16L1-knockdown cells, indicating that ATG16L1 affected the efficiency of autophagic targeting and the total cellular autophagy level.

Transfected full-length C3 could bind to ATG16L1 (**Figure 4B**). Hence, we explored if full-length C3 without deposition could also promote autophagy. C3 was transfected into A549 cells before *S. aureus* infection. Compared with C3 deposited on S. aureus, endogenous C3 lost the ability to regulate autophagy (**Figure 4F**), which suggested that extracellular cleavage of C3 by complement factors was essential for the regulation of autophagy. Taken together, these findings suggested that although both C3 deposition and full-length C3 interacted with ATG16L1, the C3 deposition on the surface of *S. aureus* rather than full-length C3 could upregulate autophagy by recruiting and interacting with ATG16L1.

### 3.5 C3 deposition facilitated an ATG16L1-dependent restriction of *S. aureus* proliferation

**Figure 5:**
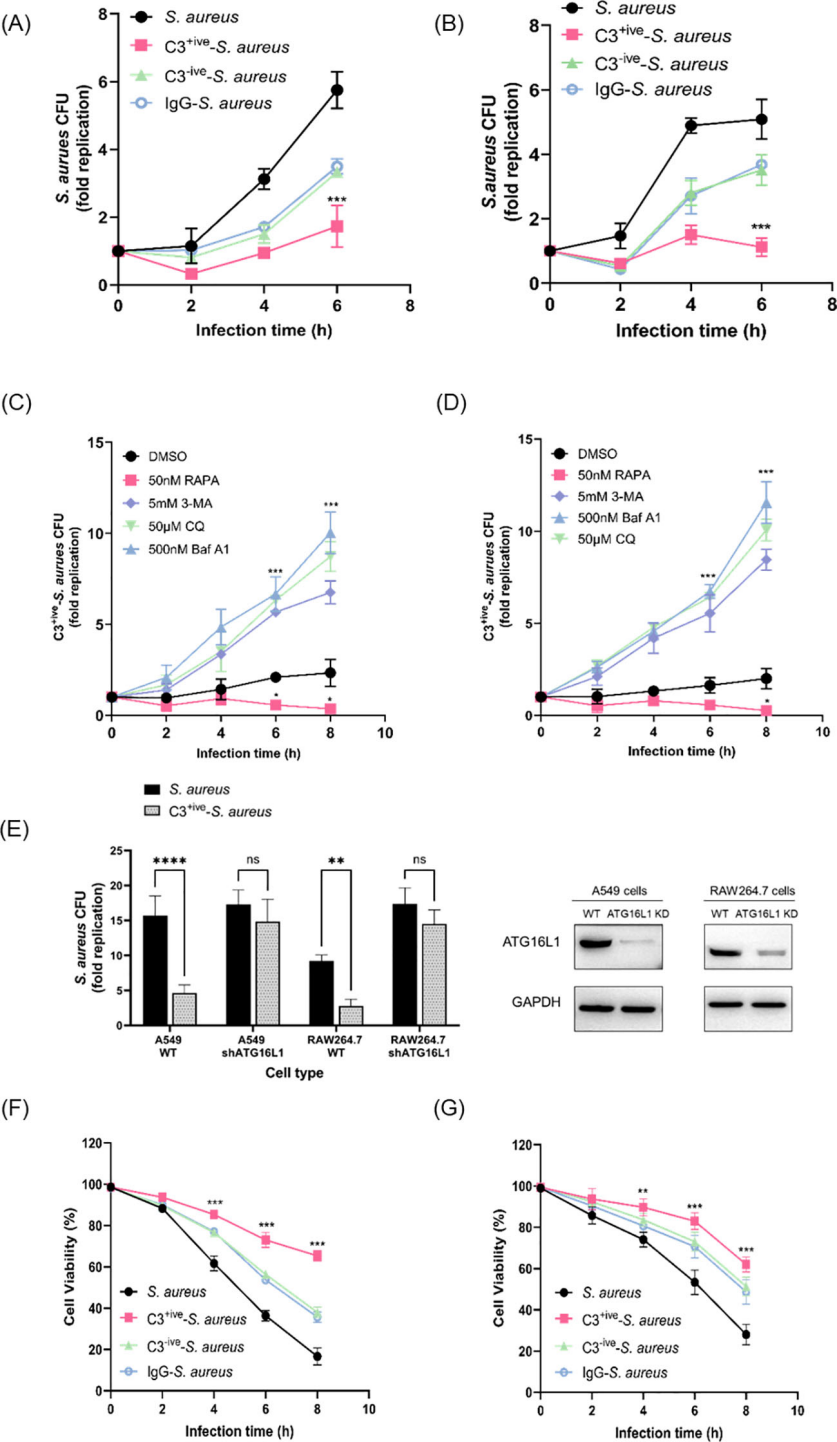
C3 coating of *S. aureus* limited intracellular proliferation through xenophagy. **(A, B)** Intracellular levels of *S. aureus*, C3^+ive^-*S. aureus*, C3^-ive^-*S. aureus* and IgG- *S. aureus* in A549 cells **(A)** and RAW264.7 cells **(B)**. After 1 h, gentamycin was added to remove extracellular bacteria, at which point the infection was recorded as 0 hpi. [T0]. Cells were then harvested and the number of intracellular bacteria was determined by colony counting on LB agar. The fold replication of *S. aureus* from each infection was the ratio of CFU/mL at different infection points to CFU/mL at T0, shown as dots, and the bar represents the mean value. **(C, D)** Levels of C3^+ive^-*S. aureus* within A549 cells **(C)** and RAW264.7 cells **(D)** upon treatment with rapamycin, 3-Methyladenine, bafilomycin A1, or chloroquine. Drug treatment of cells in conjunction with bacterial infection. The fold replication of C3^+ive^-*S. aureus* is shown as described above. **(E)** Levels of C3^+ive^-*S. aureus* within wild-type cells and ATG16L1-knockdown cells. The fold replication of C3^+ive^-*S. aureus* is shown as described above. 6 hours after changing the medium, infected cells were harvested and used for bacteria counting. **(F, G)** Viability of A549 cells **(F)** and RAW264.7 cells **(G)** after infection with *S. aureus*, C3^+ive^-*S. aureus*, C3^-ive^-*S. aureus* and IgG- *S. aureus*. Representative of n = 3 independent experiments. MOI = 50. ^ns^p > 0.05, **p ≤ 0.01, ***p ≤ 0.001, ****p ≤ 0.001.

Autophagy contributes to intracellular bacterial restriction by targeting invading agents to lysosomes for degradation. We aimed to determine if the C3 deposition facilitated autophagy-dependent restriction of internalized *S. aureus*. We normalized the amount of *S. aureus*, C3^+ive^-*S. aureus*, C3^-ive^-*S. aureus* and IgG-*S. aureus* before infecting mammalian cells, and measured the replication of bacteria after infection. The levels of internalized *S. aureus* in A549 cells (**Figure 5A**) and RAW264.7 cells (**Figure 5B**) decreased dramatically when bacterial cells were coated with C3. Meanwhile, the degree of proliferative restriction mediated by C3 was further increased by addition of an autophagy inducer, rapamycin, and was suppressed by autophagy inhibitors (3-Methyladenine, bafilomycin A1 and chloroquine) (**Figures 5C, D**). Importantly, the reduction of bacterial proliferation caused by C3 deposition no longer occur when ATG16L1 was depleted (**Figure 5E**).

**Figure 6:**
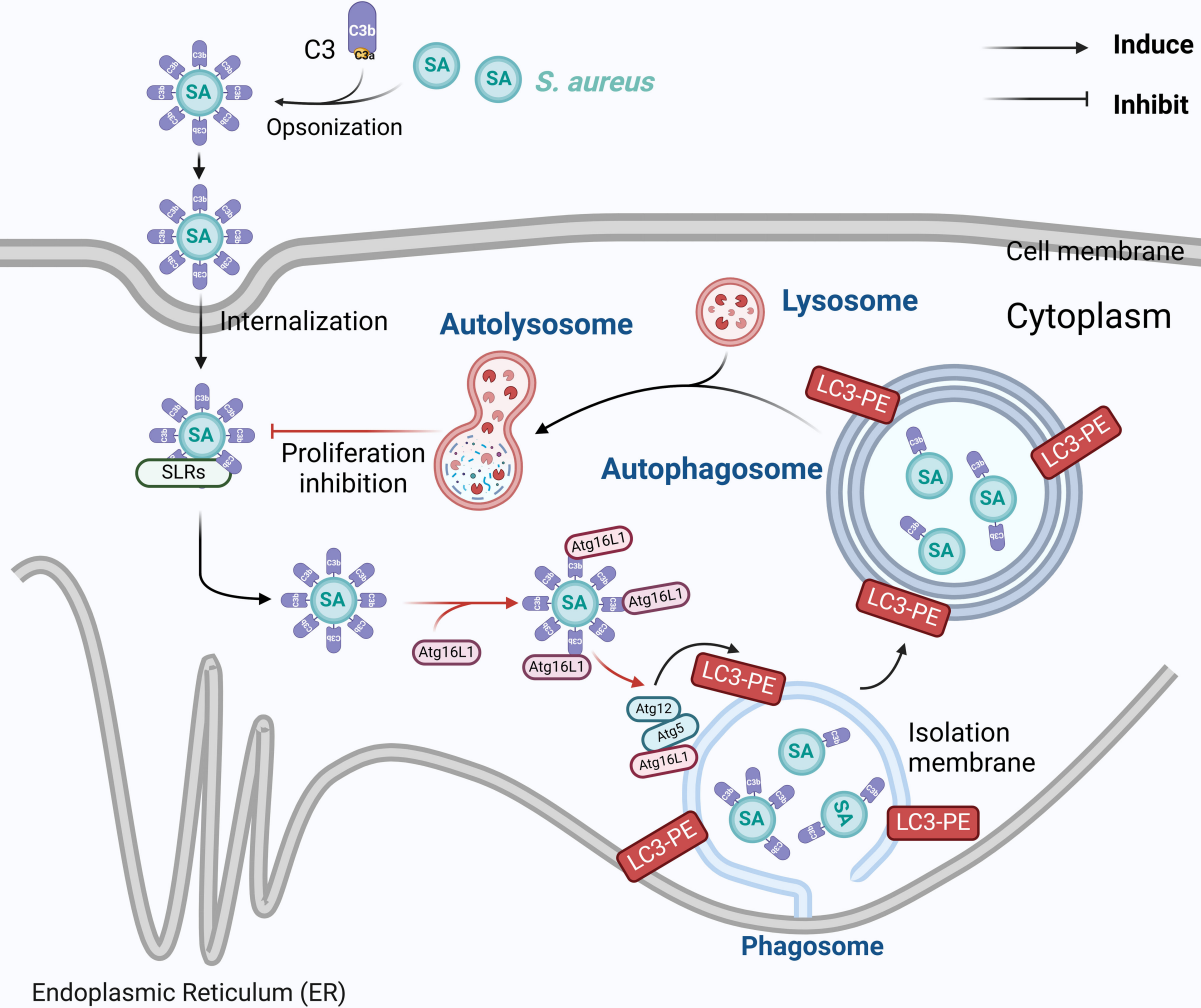
Mechanism of C3 opsonization in facilitating autophagy-dependent limitation of internalized *S. aureus* by ATG16L1.The schema was generated by using Biorender.

Next, the viability of A549 and RAW264.7 cells after infection was examined by the CCK-8 assay. Remarkably, host cells displaying highest viability were those infected by C3^+ive^-*S. aureus*, suggesting the protective role of C3 coating on host cells (**Figures 5F, G**). Meanwhile, cells infected with C3^-ive^-*S. aureus* and IgG-*S. aureus* exhibited similar viability, indicating the dominant role of C3 in preventing bacterial proliferation and protection host cells. Taken together, these results demonstrated that the C3 deposition facilitated autophagy-dependent limitation of internalized *S. aureus* by ATG16L1 and had a protective effect on cells (**Figure 6**).

## 4 Discussion

Hosts recognize bacteria through a complement system, the process that C3 deposited on bacteria is inevitable. However, there is still a lack of knowledge about intracellular C3 for eliminating internalized bacteria. *S. aureus* has a broad host range and can enter cells through various mechanisms, including phagocytosis and endocytosis (Bayles et al., 1998; Moldovan and Fraunholz, 2019). In phagocytosis, immune cells such as neutrophils engulf bacteria by forming a phagosome, which fuses with a lysosome to form a phagolysosome (Urban et al., 2006). The acidic environment and lysosomal enzymes in the phagolysosome contribute to the degradation of bacteria (Gibson et al., 2021). In endocytosis, *S. aureus* enters non-phagocytic cells by exploiting the receptors and pathways of host cells (Bhavsar et al., 2007). This process is often mediated by proteins on the bacterial surface such as clumping factor B and fibronectin-binding proteins, which bind to host cell receptors and trigger endocytosis (Bravo-Santano et al., 2018; Fraunholz and Sinha, 2012). Deposition of C3 onto the surface of bacterial pathogens has a critical impact on the innate and adaptive immune responses of the host (Dunkelberger and Song, 2010). We showed that the C3 continued to be found on *S. aureus* surfaces after entering phagocytic cells and non-phagocytic cells. The covalent bonds between C3 and *S. aureus*, such as thioester bonds, may help C3 to be stable on the bacterial surface through phagocytosis and endocytosis (Levashina et al., 2001).

Several studies have emphasized the function of LAP in autophagy machinery as well as the links between the presence of LC3 on bacteria-containing phagosomes and antimicrobial autophagy (Prajsnar et al., 2021; Vozza et al., 2021). Our results indicated that *S. aureus* and C3^+ive^-*S. aureus*-initiated autophagy in A549 cells (non-phagocytic). The main autophagy machinery caused by bacteria of the genera *Salmonella*, *Listeria*, and *Shigella* in other types of non-phagocytic cells (e.g., HeLa, HCT116) has also been shown to involve xenophagy (Ammanathan et al., 2020; Thurston et al., 2009, 2012).

*S. aureus* has been shown to elicit autophagy, but the autophagic response to C3^+ive^-*S. aureus* in non-phagocytes has not been studied. Once inside a cell, *S. aureus* can survive and replicate by modifying signaling pathways in host cells and avoiding detection by the immune system (Galluzzi and Green, 2019). C3 within cells is involved in phagocytosis, cell signaling, and regulation of the immune response (Kulkarni et al., 2019). Its role in promoting opsonization by phagocytic cells is well documented, but our findings demonstrated that the C3 deposition impacted the infection of non-phagocytic (e.g., A549) cells by internalized *S. aureus*. C3 deposition upregulated autophagy and increased autophagic flux by recruiting and interacting with ATG16L1. Similar to our results, data from other studies have identified a role for cytosolic proteins interacting with C3 (e.g., Sequestosome 1 (SQSTM1/p62), NOD-like receptor thermal protein domain associated protein 3 (NLRP3), macrophage-1 antigen (ASC)) in activating autophagy or LAP (Gluschko et al., 2018; Kim et al., 2016; Tsai et al., 2018).

Additionally, we discovered that only C3 protein deposited on bacterial surfaces can enhance autophagy during infection as C3 transfected in the absence of bacteria failed to do so. Based on these results, it can be inferred that the enzymatic cleavage of C3 is a prerequisite for its ability to modulate autophagy. C3 undergoes a series of processes to deposit on bacterial surfaces. Upon activation, C3 is cleaved into two fragments: C3a and C3b. The latter is a larger fragment that can bind covalently to microbial surfaces. C3b is involved in opsonization, phagocytosis, and complement-mediated cell lysis. If C3b goes through a further proteolytic cleavage, smaller fragments (e.g., C3c, C3d) are generated which help to amplify the immune response (Lambris, 1988). The complement cascade and changes in complement proteins are complex and rapid. Hence, knowing the details of C3 opsonization and identifying the C3 fragments that enter cells eventually is difficult (Rooijakkers et al., 2009). In our experiments, numerous attempts have been made to identify the critical C3 fragments that exert a functional role within the cell. The eukaryotic expression of full-length C3 within cells can partially elucidate, but does not comprehensively explain, the impact of extracellular complement cascade reaction on the modulation of intracellular autophagy.

Bacteria have evolved mechanisms to escape from autophagy. Multiple lines of evidence suggest that specific bacterial effectors/toxins inhibit this defense process (Knodler and Celli, 2011). We revealed that internalized *S. aureus* may lose toxins/enzymes to inhibit C3 deposition because surprisingly high targeting by autophagy was noted in A549 cells. *S. aureus* has a great capacity for mutation. The α-toxin secreted by methicillin-resistant *Staphylococcus aureus* (MRSA) interferes with autophagosome maturation and blocks the fusion of autophagosomes with lysosomes (Maurer et al., 2015).

Understanding the mechanisms that regulate the targeting of internalized bacteria to the autophagy system can provide insights into therapeutic strategies. Several cytosolic adaptor molecules, such as nuclear dot protein 52, galectin-8, p62, optineurin, and NODs (e.g., NOD1 and NOD2), have been shown to contribute to xenophagy induction by favoring the recruitment of autophagic machinery to the vicinity of the bacteria to be engulfed (Jakopin, 2014; Lin et al., 2020; O'Seaghdha and Wessels, 2013; Ravenhill et al., 2019; Zheng et al., 2009).

Intriguingly, various cellular immune responses can employ autophagy to restrict bacterial growth, whereas some do not rely entirely on the full autophagic flux (Masud et al., 2019). We found that the C3 deposition protected host cells against infection by restricting intracellular staphylococcal growth in an ATG16L1-dependent manner. Our results support a direct autocrine role or paracrine role of ATG16L1, which is necessary for protection against MRSA strains through the release of a disintegrin and metalloproteinase domain-containing protein 10 exosomes to bind the α-toxin extracellularly (Keller et al., 2020).

In summary, our study demonstrated that the C3 coating on the surface of *S. aureus* had an important role in host-directed antibacterial autophagy. C3 may be a universal regulator of xenophagy through its interaction with ATG16L1 intracellularly and subsequent upregulation of autophagy. Notably, C3 must be activated before upregulation of autophagy (though unsliced C3 is expected to interact with ATG16L1). The importance of this defense mechanism was highlighted further by the findings that the *S. aureus* ATCC 29213 strain did not evolve mechanisms to degrade its C3 coating rapidly and escape C3-mediated autophagy restriction: this phenomenon could provide a novel strategy to limit internalized bacteria.

## Data Availability

The original contributions presented in the study are included in the article/supplementary material. Further inquiries can be directed to the corresponding authors.
