# Supplementary material for: Complement C3 deposition restricts the proliferation of internalized Staphylococcus aureus by promoting autophagy
Source: Front Cell Infect Microbiol. 2024 Sep 6;14:1400068. doi: 10.3389/fcimb.2024.1400068 (PMC11412942; doi:10.3389/fcimb.2024.1400068)
Supplement: Supplementary file 1 [file Table1.docx]

<https://www.jianguoyun.com/c/sd/18f61b0/4ebfe9d9d0e5f738#from=https%3A%2F%2Fwww.jianguoyun.com%2Fc%2Fsd%2F18f61b0%2F4ebfe9d9d0e5f738>
